# Household Food Insecurity, Underweight Status, and Associated Characteristics among Women of Reproductive Age Group in Assayita District, Afar Regional State, Ethiopia

**DOI:** 10.1155/2018/7659204

**Published:** 2018-05-14

**Authors:** Jemal Abdu, Molla Kahssay, Merhawi Gebremedhin

**Affiliations:** ^1^Department of Public Health, Samara University, Afar, Ethiopia; ^2^School of Public Health, Haramaya University, Harar, Ethiopia

## Abstract

**Background:**

Poor nutritional status of women has been a serious problem in Ethiopia. Rural women are more likely to be undernourished than urban women. Afar region is the most likely to be undernourished (43.5%). Despite the humanitarian and food aid, food insecurity and maternal underweight are very high in the region. Household food insecurity is not adequately studied in Afar region. The aim of this study was to assess the prevalence of household food insecurity and underweight status and its association among reproductive age women.

**Method:**

The study was conducted in Assayita district in June 2015. Community-based cross-sectional study design was used among nonpregnant women. Household data was collected using structured questionnaire. Multistage cluster sampling procedure was applied. Two pastoral and two agropastoral Kebeles have been selected by simple random sampling. Systematic random sampling was used to select respondents. The total sample size was 549 households. Household Food Insecurity Access Scale (HFIAS) and anthropometric data were used to determine food insecurity and underweight, respectively. Multivariate regression models were used to measure associations.

**Results:**

Prevalence of HFIAS was 70.4 with a mean of 7.0 (3.6 ± SD); 26.1%, 30.20%, and 14.1% were mild, moderate, and severe food insecurity, respectively. Underweight prevalence (BMI < 18.5) was 41.1% with prevalence of mild, moderate, and severe underweight being 34.5%, 3.9%, and 2.7%, respectively. Age, parity, and having >2 children below five years of age were statistically associated with household food insecurity and maternal underweight.

**Conclusion:**

Household food insecurity and maternal underweight were very high. Age, parity, and having ≥2 children below five years of age were associated with household food insecurity. Maternal underweight was associated with maternal age, marital status, parity, number of children below 5 years, household food insecurity, and vocation of the respondents.

## 1. Background

Food insecurity is a state or a condition in which people experience limited or uncertain physical and economic access to safe, sufficient, and nutritious food to meet their dietary needs or food preferences for a productive, healthy, and active life [1]. Food security, on the other hand, is achieved when all people, at all times, have physical, social, and economic access to sufficient, safe, and nutritious food that meets their dietary needs and food preferences for an active and healthy life [2]. Food insecurity is a major public health problem in both developing and developed nations. However, the proportion of undernourished people remains highest in sub-Saharan Africa [3, 4].

Ethiopia, one of the most food-insecure countries in Africa, has long history of famines and food shortages. More than half of the African's food-insecure population lives in Ethiopia and six other countries [5]. The nutritional status of a mother is important, both as an indicator of her overall health and as a predictor of pregnancy outcome for both mother and child [6]. The proportion of women who are malnourished in selected sub-Saharan African countries for which a DHS was recently conducted ranges from 7 to 37%. Ethiopia has highest proportions of undernourished women [7]. The national prevalence of maternal BMI < 18.5 was 26% with 40% distribution in Afar region [8].

Household food insecurity has been associated with several health and nutrition outcomes [9]. Women's nutrition affects a wide range of health and social issues, including family care and household food security [10]. Food insecurity and undernutrition in adolescent and pregnant women, compounded with gender discrimination, lead to an intergenerational cycle of nutritional problems [11]. One consequence is lowering of birth weight due to malnutrition in pregnancy, which perpetuates malnutrition between generations [7].

Ethiopian projection/forecasting for 2016 indicates that 0.4 and 1.7 million people will face severely and moderately acute undernutrition. Climatic shocks greatly affecting successive harvests and high food price inflation have combined to drive food insecurity and undernutrition significantly higher [12]. Pastoralists and agropastoralists make up nearly 15% of Ethiopia's total population and are among the poorest [13]. Ethiopia's pastoralists remain at the margins of national economic life. However, pastoral women are “doubly marginalized,” since they experience the discrimination and marginalization [14].

Afar regional state is one of the least developed of the nine regions in Ethiopia and is also the major pastoralist region of the country [15]. The region is also recognized as being hotspot for combination of high food insecurity, moderate-to-high malnutrition rates, and rapid onset of emergencies like epidemic outbreaks, floods, or conflicts [16].

Poor nutritional status of women has been a serious problem in Ethiopia for many years and requires greater multisectoral efforts [9, 17]. Rural women are more likely to be undernourished than urban women, and those residing in the Afar region are the most likely to be undernourished (43.5%) of any region [17]. The relationship between household food insecurity and nutritional status of women in Afar is not well recognized; hence, the objective of this study is to verify the prevalence of household food insecurity and maternal nutritional status in Assayita district, Afar region, Ethiopia.

## 2. Methods

The study was conducted in June 2015 in Assayita zone, Afar regional state, which is located 650 km away from Addis Ababa, the capital of Ethiopia. Based on the 2007 Census Result of the Central Statistical Agency of Ethiopia (CSAE), the total population of Afar region was 1,411,092, consisting of 786,338 men and 624,754 women. Rural inhabitants constitute 1,222,369 (86.6%) of the total population. 67.3% of inhabitants fall into the lowest wealth quintile; adult literacy for men is 27% and it is 15.6% for women [18]. Assayita is one of the largest districts which has thirteen Kebeles; of which two are urban, six are pastoral, and five are agropastoral Kebeles. Total population of the district was 47,210. Of the total population, 31,162 (66%) live in rural areas and the rest, 16,048 (34%), live in urban areas [18]. The district has four clinics, three health posts, and one health center [19].

**Figure 1:**
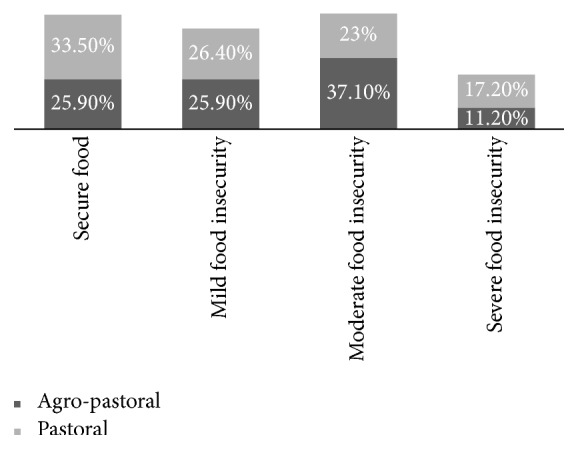
Percentage of households in each category of food security for agropastoral and pastoral households, Assayita district, June 2015 (*n* = 490).

A community-based cross-sectional study design was applied and the source population was all households with reproductive age women, while study population was households of randomly selected agropastoral and pastoral community (Figure 1). Households with at least one reproductive age woman were included. However, if more than one eligible woman was available in one household, the one who is responsible for family care and/or is head was considered for this study.

Sample size was computed using single population proportion formula assuming a marginal error of 5% and 95% confidence interval. During sample size determination, prevalence of undernourished women and national prevalence for food insecurity were taken into consideration and the prevalence that yields the maximum sample size was taken as final sample.

Besides this, 35% undernourished prevalence rate from national food insecurity survey [17] with 5% nonresponse rate and design effect of 1.5 gives us a maximum sample size of 549. The sample was distributed across the selected Kebeles proportional to their household size. With regard to sampling procedures, first multistage stratified sampling procedure was deployed to get a representative data. Two pastoral and two agropastoral Kebeles were selected using simple random sampling. Systematic random sampling was used to identify respondents and probability proportionate to size (PPS) technique was applied.

Data was collected through interviews and anthropometric measurements. During interview, structured questionnaire consisted of socioeconomic and demographic characteristics and frequency of 24-hour dietary recall and household food insecurity measurements were used. The questionnaire was initially prepared in English and then translated into Amharic. Six experienced data collectors who had Diploma certificate in health and were able to speak the local language fluently collected the data. Meanwhile, two supervisors from the district health office were involved in supervising the overall data collection process.

Household Food Insecurity Access Scale (HFIAS) was used to create a continuous numeric food insecurity “score,” which can then be compared to established cut-points to categorize the level of food insecurity experienced by the household. Nine-item questionnaire with three domains of food insecurity, anxiety/uncertainty about the household food supply, insufficient quality of food (including variety and food preferences), and insufficient food intake and its physical consequences, was used.

The participants' responses indicate a frequency of occurrence of the following: never, rarely (1 to 2 times), sometimes (3 to 10 times), and often (>10 times) for each of the questions over the previous 30 days. This was then used to calculate HFIAS scores. HFIAS scores range from 0 to 27, with a higher score indicating greater food insecurity [20]. The last three questions of the HFIAS were used to calculate the Household Hunger Scale (HHS). The three questions inquired about whether participants “had no food in the house,” “went to sleep hungry,” or “lacked food for 24 hrs.” The household score recodes the responses to each frequency-of-occurrence question from three frequency categories (“rarely,” “sometimes,” and “often”) into two frequency categories (“rarely or sometimes” and “often”). Each household will have score between 0 and 6. These values are then used to generate the household indicators which in turn are categorized into little to no hunger (0-1) in the household, moderate hunger (2-3) in the household, and severe hunger (4–6) in the household [21].

Data on household dietary diversity was collected using a 24-hour recall method and information was entered into the Household Dietary Diversity Score (HDDS) sheet. The HDDS captures dietary diversity in a normal 24-hour period by the household as a whole and not a single member. Food consumed outside the home which was not prepared in the home was not included. A set of 12 food groups were used to guide the scoring as per the food items consumed, with 1 being the minimum score and 12 being the maximum score [22].

To determine the impact of household food insecurity on nutritional status of reproductive age women's weight, height measurements were taken from all study subjects. Weight was measured to the nearest 0.5 kg using a weight measurements scale. Height was measured to the nearest centimeters also using tap meter; the scales were calibrated after each session of measurements. Malnutrition in women was assessed using the body mass index (BMI), which is defined as a woman's weight in kilograms divided by the square of her height in meters (BMI = kg/m^2^). A BMI below 18.5 among nonpregnant, nonlactating women indicates chronic energy deficiency or undernutrition. When BMI is above 25, women are considered overweight [6]. Underweight prevalence (BMI < 18.5 kg/m^2^) was further categorized by WHO standards for mild (BMI: 18.5–17 kg/m^2^), moderate (BMI: 16.99–16.00 kg/m^2^), and severe (BMI: < 16 kg/m^2^) underweight [23].

Household food insecurity status and underweight status among women of reproductive age were considered as dependent variables, whereas sociodemographic characters, height, weight, and BMI were our independent variables.

To ensure quality of data, structured questionnaire was employed to attain the required information after getting written and verbal consent from the respondents. The data collectors and supervisors were trained on objectives of study sampling procedures, techniques of interviews, and data handling. The questionnaire was pretested in a community similar to the study population and the necessary modification was made. The supervisors and principal investigator were closely following the day-to-day data collection process and ensured completeness and consistency of questionnaire administered each day. Statistical software was used to analyze data. The data was entered using Epi Info version 7 and analysis was done using Statistical Package for Social Sciences (SPPS version 21).

Descriptive statistics were tabulated to describe the characteristics of households in each level of food security, as well as the nutritional outcomes associated with food security. For variables expressed as percentages or proportions, chi-square test was used to assess differences between food security classifications. Multiple binary logistic regression models were used to quantify the association between household food security and nutritional outcomes among reproductive age women.

Ethical clearance was obtained from ethical review committee of College of Health Sciences, Addis Ababa University. An official letter was also obtained from Afar Regional Health Bureau and district health office. Similarly, written consent was obtained from interviewee before proceeding to data collection. All information that was obtained from the individual was treated confidentially.

In this study, underweight was defined as BMI < 18.5 kg/m^2^; normal weight was defined as BMI ≥ 18.5 and <25 kg/m^2^ [23]. Food-secure household was defined as the household that experiences none of the food insecurity (access) conditions or just experiences worry but rarely. Mildly food-insecure household is defined as the household that sometimes or often worries about not having enough food and/or is unable to eat preferred foods and/or eats a more monotonous diet than desired and/or some foods considered undesirable but only rarely but it does not cut back on quantity and does not experience any of three most severe conditions.

Moderately food-insecure household is considered so if it sacrifices quality more frequently by eating a monotonous diet or undesirable foods sometimes or often and/or has started to cut back on quantity by reducing the size of meals or number of meals rarely or sometimes. But it does not experience any of the three most severe conditions. Severely food-insecure household is the household that is forced to cut back on meal size or number of meals often and/or experiences any of the three most severe conditions (running out of food, going to bed hungry, or going a whole day and night without eating), even as infrequently as rarely this is assumed as. Dietary diversity is the number of different foods or food groups consumed over 24-hour period.

## 3. Results

A total of 490 households with 89.3% response rate were assessed in this study. The majority (93.3%) of the household heads interviewed were married. 269 (54.9%) of the respondents were in the age group of 30–39 years and the mean maternal age was 32.4 (±6.7 SD). The mean family size was 6.3 (±2.3 SD). More than two-thirds (63.1%) of households had 4–7 family members. 445(90.8%) of mothers were illiterate or had no formal education. Of the total respondents, 251 (51.8%) and 239 (48.2%) were agropastoralists and pastoralists, respectively.

The mean Household Food Insecurity Access Scale score was 7.0 (±3.6 SD). 345 (70.4%) of respondents were found with food insecurity. Nearly one-fifth of households, 69 (14.1%), experienced severe food insecurity, while 148 (30.2%) and 128 (26.1%) households had moderate and mild food insecurity, respectively. This study also indicated that 11.2% of agropastoralists and 17.2% of pastoralists experienced severe food insecurity.

Nearly three-quarters of households (70.8%) reported that they worried about the availability of enough food in the household, and more than two-thirds (68.6%) reported the absence of the preferred food to eat and 63.3% of respondents reported that they consumed a limited variety of food in the past 30 days. 7.8% of households reported eating fewer meals in a day or had family members that go to sleep at night hungry or complained of no food to eat in the last 30 days prior to the survey.

Households were asked about their coping strategies when faced with food insecurity; 84 (17.1%) reported reducing the amount of consumed diet per meal, 90 (18.4%) have cut the number of meals consumed per day, 22 (4.5%) shift to less quality/inexpensive foods, 53 (10.8%) reduce nonfood expenditure, 167 (34.1%) households receive food or cash aid, and 167 (15.1%) sell any household asset.

The study reveals that the mean dietary diversity score of households was 4.7 ± 1.9 (SD). Using this mean score, households were categorized into three equal parts; thus 258 (52.7%) households had consumed 5 or less food groups (poor dietary diversity), while 67 (13.7%) had consumed 7 or more food groups (high dietary diversity). Milk and milk products (94.5%), cereals (85.3%), sugar (77.8%), and miscellaneous foods like tea and coffee (77.6%) were the most commonly consumed food groups, while fish (0%), fruits (2.0%), egg (2.9%), vegetables (2.9%), and root (9.2%) were the least consumed food groups.

Underweight status in reproductive age women was assessed using BMI and the prevalence of underweight (BMI < 18.5) was 41.1% among nonpregnant women (*n* = 490), with percentage of mild, moderate, and severe underweight being 34.5%, 3.9%, and 2.7%, respectively. The mean weight, height, and BMI were 42.49 kg, 1.51 M, and 18.53 kg/m^2^, respectively.

In the binary logistic regression analysis, maternal age, maternal educational status, parity, and having more than two children below five years of age were found to be associated with increased HFIAS scores. 20–29-year-old women had less odds of food insecurity compared to 40–49-year-old women (COR = 0.35, 95% CI: 0.18, 0.69). Household heads with no formal education had more than 3 times the odds of food insecurity when compared with household heads with primary education (COR = 3.10, 95% CI: 1.64, 5.69).

Similarly, households with at least five children (parity 5+) had higher odds of food insecurity compared with those who have never had a child (parity 0) (COR = 10.56, 95% CI: 1.62, 68.88). On the other hand, households with ≥2 children below five years of age had significantly higher odds of food insecurity when compared with those who had no children below five years of age (COR = 5.96, 95% CI: 3.55 to 10.01).

The secondary, analytical objectives were to assess hypothesized predictor variables of underweight. Binary logistic regression explored predictor variables of underweight (BMI < 18.5), where age of the female respondent, marital status of women, family size, parity, children below 5 years of age, food insecurity, and vocation were significant predictors of underweight (Table 2).

This result has shown that 30–39-year-old women had less odds of underweight compared to 15–19-year-old women (COR = 0.51, 95% CI: 0.27, 0.96). Women who have never been married had more than 3 times odds of underweight compared with married women (COR = 3.61, 95% CI: 1.68, 7.75). The number of children ever born (parity) was another important factor found to significantly affect women's nutritional status.

Similarly, women with ≥7 family members were more than 2 times (COR = 2.91, 95% CI: 1.38, 6.13) likely to be underweight compared with women with 1–3 family members. Women with two or more children below five years of age had significantly higher odds of underweight compared with those who had no children below five years of age (COR = 4.99, 95% CI: 3.07 to 8.13). On the other hand, women with severe food insecurity were more than 14 times more likely to be underweight when compared with women who were food-secure (COR = 14.40, 95% CI:.7.14, 29.06). Pastoral women were 1.66 times more likely to be underweight when compared with agropastoral women (COR = 1.66, 95% CI: 1.16, 2.39).

In multiple binary logistic regression analysis, maternal age, number of children ever born (parity), and having ≥2 children below five years of age were significantly and independently associated with food insecurity. 20–29-year-old women and 30–39-year-old women had less odds of food insecurity compared to 40–49-year-old women (AOR = 0.34, 95% CI: 0.12, 0.95; and AOR = 0.34, 95% CI: 0.14, 0.79, resp.). Similarly, households with at least five children (parity 5+) had significantly higher odds of food insecurity compared with those who have never had a child (parity 0) (AOR = 10.76, 95% CI: 1.38, 84.28). On the other hand, households with one child below five years of age had significantly higher odds of food insecurity when compared with those who had no children below five years of age (AOR = 2.03, 95% CI: 1.03 to 3.98).

Multiple logistic regression models were used to predict the probability of underweight among women of reproductive age. Variables that were significantly associated with female underweight included maternal age, marital status, parity, number of children below 5 years of age, food insecurity status, and vocation of the respondents. Women who belong to the age group of 15–19 years compared with those who belong to the age group of 30–39 years had less odds of underweight (AOR = 0.10; 95% CI: 0.04, 0.27). Relative to the odds of underweight among married women, women that were never married had more than 8 times higher odds of being underweight (AOR = 8.58, 95% CI: 2.98, 24.73).

Unlike women with ≥5 children ever born (parity), women with 1-2 children ever born (parity) were less likely to be underweight (AOR = 0.28, 95% CI: 0.09, 0.83). Women with two or more children below five years of age had significantly more than 9 times higher odds of underweight compared with those who had no children below five years of age (AOR = 9.27, 95% CI: 3.36, 25.59).

Another important factor found to significantly affect women's nutritional status was food security categories and multiple logistic regression analysis found that severely food-insecure women were more than 6 times likely to be underweight when compared with food-secure women (AOR = 6.99, 95% CI: 2.66 to 18.38). On the other hand, pastoral women were more than 2 times likely to be underweight when compared with agropastoral women (AOR = 2.14, 95% CI: 1.33 to 3.44).

## 4. Limitation of the Study

This study is exposed to a recall bias when asked on 24-hour recall and what happened 4 weeks/30 days back to complete HDDS and HFIAS/HHS questions. In calculating the HDDS to capture dietary diversity in a normal 24-hour period by the household as a whole, foods consumed outside the home which were not prepared in the home were not included and this can underestimate HDDS in situations where consumption of foods not prepared in the household outside the home is common, but in this study such situations were not common.

## 5. Discussion

In this study, the prevalence of household food insecurity and that of maternal nutritional status were 345 (70.4%) and 41.1%, respectively. Maternal age, number of children ever born (parity), having ≥2 children below five years of age were significantly associated with household food insecurity. Correspondingly, marital status, maternal age, parity, number of children below 5 years of age, food insecurity, and vocation of respondents were found to be important predictors of maternal underweight status.

Underweight is affected by both health and food security status of the individual. Thus, the evaluation of undernutrition needs to be seen in light of these two pillars [24]. In this study, among nonpregnant women (15–49), the rate of underweight was 41.1%, which is higher than EDHS 2011 national rates of 27% but very similar to the rate specific to Afar region (43.5%) found in EDHS 2011 [17]. This study found a high rate of underweight women with rates similar to EDHS 2000 national survey for Afar region [25].

In multiple logistic regressions analysis, six main risk factors associated with underweight status among women have been identified. Variables that were significantly associated with female underweight included maternal age, marital status, parity, number of children below 5 years of age, food insecurity status, and vocation of the respondents. All were positively correlated with underweight among women. This study was not able to observe a significant association between maternal education level and underweight status. Previous studies have reported similar results in Vietnam [26]. However; some studies in Ethiopia, in Africa, and in Guatemala reported different results [17, 25, 27]. This lack of significant effect in the current study may be due to a small number of educated household heads relative to household heads with no education.

Another important determinant of the nutritional status of women is maternal age. Women who belong to the age group of 30–39 years are less likely to be underweight than women who belong to the age group of 15–19 years, which is very similar to other studies' results [17, 25, 27]. Lack of awareness in adolescent women about their own health and nutritional status could be a reason associated with their poor nutritional status. Moreover, 15–19-year-old women need adequate nutrients to support fast physical, mental, and emotional growth [24]. Women who have never been married are more likely to be underweight than married women. Other studies have found similar results [25, 27, 28]. The reason could be due to cultural values and poor decision-making autonomy; women have little access to higher education and high- and middle-income jobs. Hence, many women are dependent on their partners and thus may not be able to get adequate nutrition if they have no partner [24].

This study indicated that women with two or more children below five years of age had significantly higher odds of underweight compared with those who had no children below five years of age. This finding is also consistent with other studies [26]. Regarding parity, women with 1-2 children ever born (parity) had lower odds of underweight compared with those who had 5+ children ever born (parity). Similar results were found in Ethiopia and Guatemala [25, 27, 28]. This is because of having more children below five years of age and relatively higher-level parity (more children ever born) could obligate them to take care of their children rather than protecting their own health and nutritional status, given limited household resources [24].

Household food insecurity was a significant factor associated with underweight status. Both bivariate and multivariate results confirmed that those with severely food-insecure households are at a higher risk of underweight than their food-secure counterparts. This finding is also consistent with other studies [26]. This may indicate that household food security is a precondition for daily dietary intake for all household members. The pastoral women were more likely to be underweight when compared with agropastoral women. The reason could be due to a cultural problem that limits their required dietary intake from crop products [24].

Food insecurity is one of the most crucial problems threatening millions of people in Ethiopia. The HFIAS scale measurement revealed that 70.4% of the households faced food insecurity, which was higher than findings reported from Tigray (42.7%) and Amhara (43.8%) [29, 30]. The possible justifications might be that, in most of Afar region, the Belg rains were far below average. With little pasture regeneration or refilling of water points, most areas remained in the dry season. Accordingly, livestock body conditions have deteriorated, and livestock production and productivity have declined. Unseasonal livestock migration has occurred, reducing milk access for most household members. As per the National Meteorology Agency (NMA), central and northeastern Ethiopia experienced a very poor first season (Belg, February-April) of rainfall in 2015. The reason for the decrease can be attributed to the prevailing El-Niño episode and the weakened moisture incursion from the Indian Ocean [9].

The factors with strongest effect for household food insecurity were maternal age, the number of children ever born (parity), and having ≥2 children below five years of age (Table 1). Household food insecurity studies in rural Guatemala found that rural living, low socioeconomic quintiles, and more children below 5 years of age were the major determinants of food insecurity [28].

20–29-year-old women and 30–39-year old women had less odds of food insecurity than 40–49-year old women. Women who have ≥2 children below five years of age were at a higher risk of household food insecurity when compared with households with no children below five years of age. This finding is also consistent with other studies [26, 28]. In contrast, a study done in Somalia and Oromia, agropastoralist showed that dependency ratio did not have a significant impact on the odds of food availability in the household [31]. Women who have at least five children (parity 5+) were at a higher risk of household food insecurity compared to women who have no children (parity 0). The reason could be that women who have ≥2 children below five years of age and parity 5+ belong to households with relatively poor economic status, which may also make it more difficult to obtain sufficient food [24].

## 6. Conclusion

This study concludes that the magnitude of household food insecurity (70.4%) and maternal undernutrition (41.1%) were very high. Similarly, maternal age, number of children ever born (parity), and having ≥2 children below five years of age have shown statistical significance with household food insecurity. Variables that were significantly associated with female underweight include maternal age, marital status, parity, number of children below 5 years of age, food insecurity status, and vocation of the respondents.

Policy measures directed towards the provision of family planning to reduce household size should be given adequate attention. Effort should be made to provide basic social services such as education and health to overcome immediate consequence of food insecurity and undernutrition which ultimately increase human capital and are vital in changing the lives of the poor pastoral and agropastoral communities.

## Figures and Tables

**Table 1:** Multivariate analysis on factors associated with food insecurity in households of Assayita district, Afar, Ethiopia, June 2015 (*n* = 490).

Variables	Food insecurity status		*N*	COR (95% CI)	AOR (95% CI)
	Secure (%)	Insecure (%)			
*Maternal age (years)*					
15–19	8 (1.6)	36 (7.3)	44	1.44 (0.55 to 3.76)	2.13 (0.71 to 6.36)
20–29	55 (11.2)	60 (12.2)	115	0.35 (0.18 to 0.69)	0.34 (0.12 to 0.95)
30–39	67 (13.7)	202 (41.2)	269	0.96 (0.51 to 1.83)	0.34 (0.14 to 0.79)
40–49 (Ref^*∗*^)	15 (3.1)	47 (9.6)	62	1.00	1.00
*Educational status*					
No education	121 (24.7)	324 (66.1)	445	**3.10 (1.64–5.69)** ^*∗*^	1.22 (0.51–2.93)
Primary education (Ref^*∗*^)	24 (4.9)	21 (4.3)	45	1.00	1.00
*Parity*					
0 (Ref^*∗*^)	2 (0.4)	13 (2.7)	15	1.00	1.00
1-2	55 (11.2)	35 (7.1)	90	**0.10 (0.02 to 0.46)** ^*∗*^	**0.09 (0.02 to 0.48)** ^*∗*^
3-4	85 (17.3)	91 (18.6)	176	**0.17 (0.04 to 0.75)** ^*∗*^	0.18 (0.04 to 0.89)
≥5	3 (0.6)	206 (42.0)	209	**10.56 (1.62 to 68.88)** ^*∗*^	10.76 (1.38 to 84.28)
*Number of children below 5 years of age*					
0 (Ref^*∗*^)	59 (12.0)	67 (13.7)	126	1.00	1.00
1	56 (11.4)	75 (15.3)	131	1.18 (0.72 to 1.93)	2.03 (1.03 to 3.98)
≥2	30 (6.1)	203 (41.4)	233	**5.96 (3.55 to 10.01)** ^*∗*^	1.96 (0.85 to 4.49)

Ref^*∗*^: reference category; ^*∗*^*p* value < 0.05, which was considered significant.

**Table 2:** Multivariate analysis on underweight among 15–49-year-old women in households of Assayita district, Afar, Ethiopia, June 2015 (*n* = 490).

Variables	Underweight status		*N*	COR (95% CI)	AOR (95% CI)
	Yes (%)	No (%)			
*Maternal age (years)*					
15–19 (Ref^*∗*^)	23 (4.7)	21 (4.3)	44	1.00	1.00
20–29	48 (9.8)	67 (13.7)	115	0.65 (0.33 to 1.32)	0.44 (0.17 to 1.17)
30–39	96 (19.6)	173 (35.3)	269	0.51 (0.27 to 0.96)	**0.10 (0.04 to 0.27)** ^*∗*^
40–49	34 (6.9)	28 (5.7)	62	1.11 (0.51 to 2.41)	0.78 (0.26 to 2.32)
*Marital status*					
Married (Ref^*∗*^)	178 (36.6)	279 (56.9)	457	1.00	1.00
Never married	23 (4.7)	10 (2.0)	33	**3.61 (1.68 to 7.75)** ^*∗*^	**8.58 (2.98 to 24.73)** ^*∗*^
*Family size*					
1–3 (Ref^*∗*^)	14 (2.9)	23 (4.7)	37	1.00	1.00
4–7	95 (19.4)	214 (43.7)	309	0.73 (0.36 to 1.48)	0.66 (0.16 to 2.68)
>7	92 (18.8)	52 (10.6)	144	**2.91 (1.38 to 6.13)** ^*∗*^	0.86 (0.17 to 4.27)
*Parity*					
0	10 (2.0)	5 (1.0)	15	1.32 (0.44 to 3.99)	0.76 (0.09 to 6.17)
1-2	15 (3.1)	75 (15.3)	90	**0.13 (0.07 to 0.25)** ^*∗*^	**0.28 (0.09 to 0.83)** ^*∗*^
3-4	50 (10.2)	126 (25.7)	176	**0.26 (0.17 to 0.40)** ^*∗*^	0.58 (0.26 to 1.28)
≥5 (Ref^*∗*^)	126 (25.7)	83 (16.9)	209	1.00	1.00
*Number of children below 5 years of age*					
0 (Ref^*∗*^)	30 (6.1)	96 (19.6)	126	1.00	1.00
1	29 (5.9)	102 (20.8)	131	0.10 (0.03 to 0.34)	**2.77 (1.08 to 7.07)** ^*∗*^
≥2	142 (29.0)	91 (18.6)	233	**4.99 (3.07 to 8.13)** ^*∗*^	**9.27 (3.36 to 25.59)** ^*∗*^
*HFIAS categories*					
Secure (Ref^*∗*^)	29 (5.9)	116 (23.7)	145	1.00	1.00
Mild food insecurity	45 (9.2)	83 (16.9)	128	**2.17 (1.26 to 3.74)** ^*∗*^	1.35 (0.65 to 2.82)
Moderate food insecurity	73 (14.9)	75 (15.3)	148	**3.89 (2.32 to 6.54)** ^*∗*^	**2.66 (1.27 to 5.58)** ^**∗**^
Severe food insecurity	54 (11.0)	15 (3.1)	69	**14.40 (7.14 to 29.06)** ^*∗*^	**6.99 (2.66 to 18.38)** ^**∗**^
*Vocation*					
Agropastoral (Ref^*∗*^)	88 (18.0)	163 (33.3)	239	1.00	1.00
Pastoral	113 (23.1)	126 (25.7)	(251)	**1.66 (1.16 to 2.39)** ^**∗∗**^	**2.14 (1.33 to 3.44)** ^**∗**^

Ref^*∗*^: reference category; ^*∗*^*p* value < 0.05, which was considered significant.

## Data Availability

The datasets supporting the conclusions of the study are included in the article. Any additional data will be available on request.
